# Identification of the Cinnamyl Alcohol Dehydrogenase Gene Family in *Brassica* U-Triangle Species and Its Potential Roles in Response to Abiotic Stress and Regulation of Seed Coat Color in *Brassica napus* L.

**DOI:** 10.3390/plants14081184

**Published:** 2025-04-10

**Authors:** Yiwei Liu, Ziwuyun Weng, Yuanyuan Liu, Mengjiao Tian, Yaping Yang, Nian Pan, Mengzhen Zhang, Huiyan Zhao, Hai Du, Nengwen Yin, Cunmin Qu, Huafang Wan

**Affiliations:** 1 Integrative Science Center of Germplasm Creation in Western China (Chongqing) Science City, College of Agronomy and Biotechnology, Southwest University, Chongqing 400715, China; liuyiwei1942@email.swu.edu.cn (Y.L.); wzwy18716955096@email.swu.edu.cn (Z.W.); 18865550726@163.com (Y.L.); mengjiao000527@163.com (M.T.); 15662619623@163.com (Y.Y.); p1666835803@email.swu.edu.cn (N.P.); zmz0110@email.swu.edu.cn (M.Z.); zhaohuiyan@swu.edu.cn (H.Z.); haidu81@126.com (H.D.); nwyin80@swu.edu.cn (N.Y.); 2 Academy of Agricultural Sciences, Southwest University, Chongqing 400715, China; 3 Engineering Research Center of South Upland Agriculture, Ministry of Education, Chongqing 400715, China

**Keywords:** cinnamyl alcohol dehydrogenase (CAD), U-triangle species, *Brassica napus* L., abiotic stress, seed coat color

## Abstract

Cinnamyl alcohol dehydrogenase (CAD) is essential for lignin precursor synthesis and responses to various abiotic stresses in plants. However, the functions of CAD in *Brassica* species, especially in *Brassica napus*, remain poorly characterized. In the present study, we identified a total of 90 *CAD* genes across the *Brassica* U-triangle species, including *B. rapa*, *B. nigra*, *B. oleracea*, *B. juncea*, *B. napus*, and *B. carinata*. Comprehensive analyses of phylogenetic relationships, sequence identity, conserved motifs, gene structure, chromosomal distribution, collinearity, and *cis-*acting elements were performed. Based on phylogenetic analysis, these genes were categorized into four groups, designated as groups I to IV. Most of the *CAD* genes were implicated in mediating responses to abiotic stresses and phytohormones. Notably, members in group III, containing the bona fide *CAD* genes, were directly involved in lignin synthesis. Furthermore, the expression profiles of *BnaCAD* genes exhibited differential responses to drought, osmotic, and ABA treatments. The expression levels of the *BnaCAD4a*, *BnaCAD4b*, *BnaCAD5b*, and *BnaCAD5d* genes were detected and found to be significantly lower in yellow-seeded *B. napus* compared to the black-seeded ones. This study provides a comprehensive characterization of *CAD* genes in *Brassica* U-triangle species and partially validates their functions in *B. napus*, thereby contributing to a better understanding of their roles. The insights gained are expected to facilitate the breeding of yellow-seeded *B. napus* cultivars with enhanced stress tolerance and desirable agronomic traits.

## 1. Introduction

The *Brassica* genus is a globally significant crop system, providing essential commodities such as fresh vegetables, livestock fodder, edible oil, and biodiesel feedstock [1]. The U-triangle model, encompassing three diploid ancestors (*B. rapa*, *B. oleracea*, and *B. nigra*) and three allotetraploid species (*B. napus*, *B. carinata,* and *B. juncea*), serves as a conceptual framework for investigating evolutionary trajectories and polyploidization dynamics within the genus [2]. The chromosome-scale genomes of these species have been assembled, and numerous gene families have been identified [3,4,5,6,7,8].

Rapeseed (*B. napus*, 2n = 38, AACC) is an important allotetraploid species, originating from natural interspecific hybridization between *B. rapa* (AA, 2n = 20) and *B. oleracea* (CC, 2n = 18) [2]. As a dual-purpose oilseed crop, *B. napus* provides both oil and protein resources and constitutes a critical agricultural commodity with significant global economic importance [9]. Therefore, developing *B. napus* cultivars with high quality and enhanced resistance has become a key breeding objective. Primarily, yellow-seeded *B. napus* varieties are characterized by higher oil content and lower polyphenolic substances [10]. Within the *Brassicaceae* family, reduced lignin deposition is closely associated with lighter seed coat pigmentation [11], a relationship exemplified by *B. napus*, in which lignin has been demonstrated to play a negative role in the development of yellow-seeded phenotypes [12]. Moreover, enhancing stress resistance is pivotal for mitigating the persistent threats to production, since abiotic stresses, particularly drought and osmotic stress, severely impair rapeseed yield and quality by impeding growth, pollination, and seed filling [13]. Some phytohormones are crucial in stimulating plant resistance to adverse environmental conditions [14]. Consequently, elucidating the regulatory mechanisms underlying the responses of *B. napus* to abiotic stresses and phytohormones holds substantial significance for improving the stress tolerance of this crop.

Lignin synthesis in the seed coat of *B. napus* is orchestrated by a coordinated enzymatic network, including cinnamyl alcohol dehydrogenase (CAD), *p*-coumarate-CoA ligase (4CL), and ferulate 5-hydroxylase (F5H), and others [15]. Among these enzymes, CAD catalyzes the last step in the synthesis of precursors of the H (hydroxyphenyl), G (guaiacyl), and S (syringyl) lignin monomer units, thereby modulating both lignin content and composition [16]. *CAD* genes have been identified and characterized in various plant species, such as *Arabidopsis thaliana* (thale cress) [17], *Morus alba* (mulberry) [18], *Cucumis melo* (melon) [19], *Elaeis guineensis* (oil palm) [20], *Populus przewalskii* (poplar) [21], and *Oryza sativa* (rice) [22]. In angiosperms, *CAD* genes are categorized into two primary functional types. The first category comprises bona fide *CAD* genes directly involved in lignin synthesis [23], such as *AtCAD4* and *AtCAD5* in *A. thaliana* [17], *EgCAD2* in *Eucalyptus* spp. (eucalyptus) [24], *OsCAD2* in rice [25], and *ZmCAD2* in *Zea mays* (maize) [26]. Conversely, the second category includes genes predominantly associated with plant defense mechanisms against abiotic or biotic stresses [17]. For example, the expression of *AtCAD7* and *AtCAD8* in *A. thaliana* is induced by elicitor treatments and infection with *Pseudomonas syringae* [27]; *IbCAD1* in *Ipomoea batatas* (sweet potato) is involved in both jasmonic acid (JA)- and salicylic acid (SA)-mediated damage responses, as well as abscisic acid (ABA)-mediated cold responses [28]. Despite extensive research on *CAD* genes across various species, the identification and functional analysis of the gene family in the *Brassica* U-triangle species, particularly in *B. napus*, have yet to be fully explored.

Here, we identified a total of 90 *CAD* genes in the *Brassica* U-triangle species and performed a comprehensive analysis of their phylogenetic relationships, sequence identity, conserved motifs, gene structure, chromosomal distribution, collinearity, and *cis-*acting elements. Additionally, we first analyzed the expression profiles of *BnaCAD* genes in Zhongshuang 11 (ZS11, *B. napus*) under drought, osmotic, and ABA treatments. Subsequently, we analyzed the expression levels of some *BnaCAD* genes in yellow- and black-seeded *B. napus*, revealing significant differences in their expression patterns. These findings provide a foundation for the breeding of yellow-seeded *B. napus* cultivars with enhanced stress tolerance and desirable agronomic traits.

## 2. Results

### 2.1. Identification of CAD Family Genes in the Brassica U-Triangle Species

A total of 90 CAD proteins were identified across six species in the *Brassica* U-triangle model using TBtools, including 15 in *B. rapa*, 8 in *B. nigra*, 11 in *B. oleracea*, 19 in *B. juncea*, 19 in *B. napus*, and 18 in *B. carinata* (Table S1). Reliable phylogenetic comparison of *AtCAD3 (AT2G21890.1)* homologs within the *Brassica* U-triangle species was precluded due to two evolutionary constraints, namely the accelerated divergence rates complicating ortholog identification [29] and *A. thaliana*-specific genomic reorganization resulting in the loss of *AtCAD3* via segregation distortion [30]. Consequently, the CAD proteins were classified into eight distinct subgroups (CAD1–2 and CAD4–9) based on their sequence similarity to the remaining AtCAD paralogs (AtCAD1–9, excluding AtCAD3) through BLAST analysis. Within each CAD subgroup, proteins were assigned alphabetical suffixes (a, b, c, etc.) based on the ascending numerical order of chromosomal locations, with lower numerical values corresponding to the earlier alphabetical letters. As shown in Table S1, the number of amino acid residues of the 90 CAD proteins ranged from 263 (BolCAD7b) to 631 (BjuCAD1a). The molecular weights (MWs) of these proteins varied from 28.59 kDa (BolCAD7b) to 68.69 kDa (BjuCAD1a), with an average of 43.00 kDa. The isoelectric point (pI) ranged from 5.23 (BraCAD4b) to 9.27 (BjuCAD1a). Notably, BjuCAD1a is the only member predicted to localize to the cytoplasm or chloroplasts, whereas all other CAD proteins are cytoplasmic.

### 2.2. Phylogenetic Analysis of CAD Proteins in A. thaliana and the Brassica U-Triangle Species

**Figure 1:**
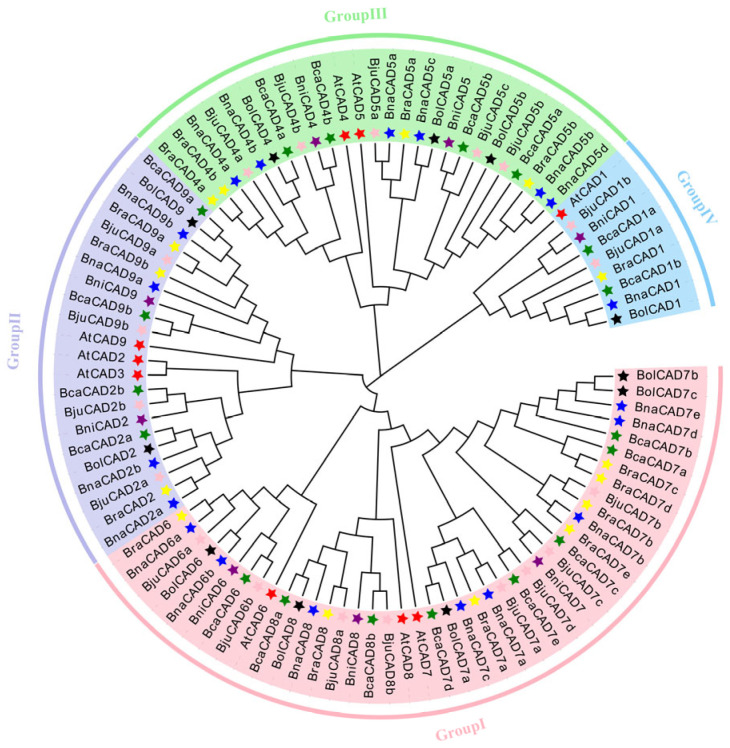
Phylogenetic tree of the CAD protein family from *A. thaliana* and the *Brassica* U-triangle species. The CAD family is divided into four groups (Groups I–IV), which are represented in pink, purple, green, and blue, respectively. Species are denoted as follows: *A. thaliana* (red star), *B. napus* (blue star), *B. rapa* (yellow star), *B. juncea* (pink star), *B. oleracea* (black star), *B. carinata* (green star), and *B. nigra* (purple star).

It was revealed that the 99 CAD proteins (9 from *A. thaliana* and 90 from the U-triangle species) could be classified into four distinct groups, designated as Group I to Group IV (Figure 1). Notably, each group contained representatives from all seven species. Among these groups, the 26 proteins in Group III are most likely involved in lignification, as they are orthologous to AtCAD4 and AtCAD5, which have been well characterized as *CADs* implicated in lignin synthesis [17]. Group I covered 42 proteins from the CAD6, CAD7, and CAD8 subgroups. Group II contained 22 proteins consisting of two subgroups (CAD2 and CAD9). Group IV included nine proteins from the CAD1 subgroup. Given the conservation of the CAD function across these subgroups, most CAD proteins are likely to participate in responses to abiotic stresses and phytohormones.

### 2.3. Multiple Sequence Alignment of CAD Proteins in A. thaliana and the Brassica U-Triangle Species

**Figure 2:**
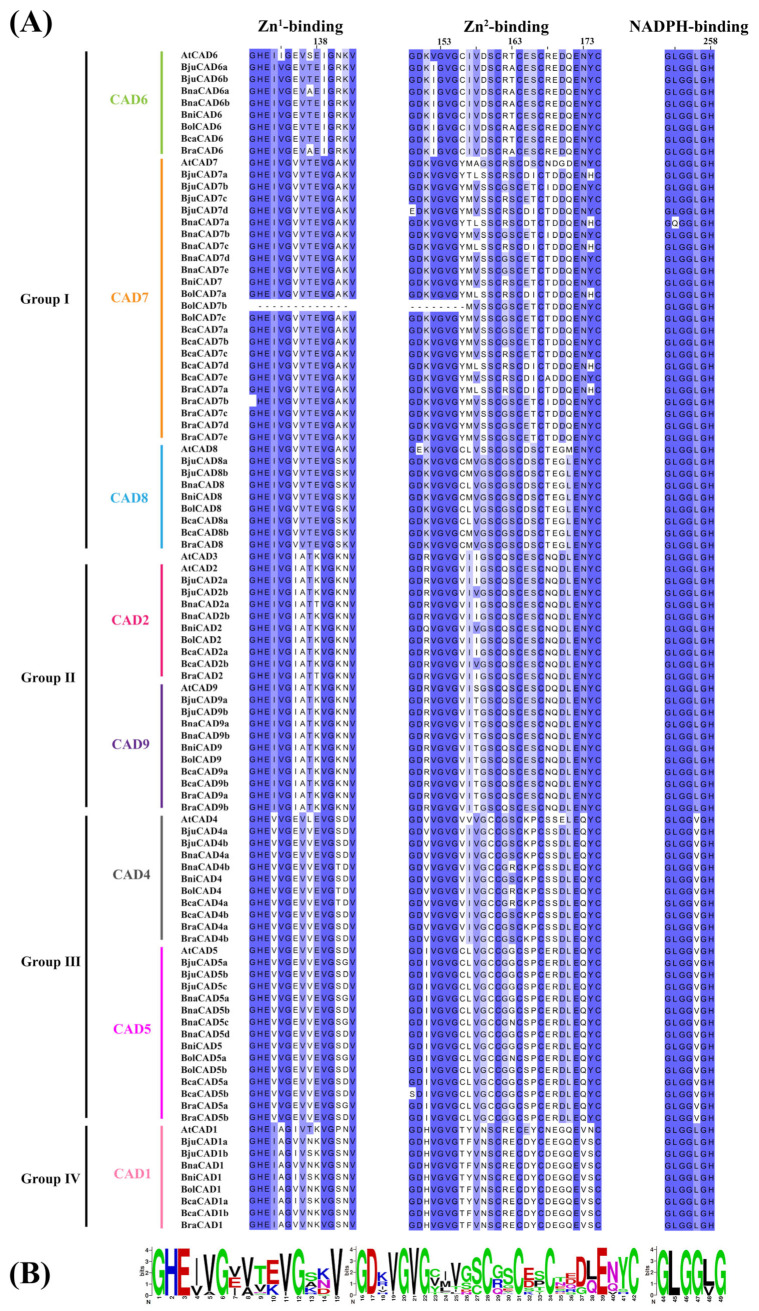
Multiple sequence alignment of CAD proteins in *A. thaliana* and the *Brassica* U-triangle species. (**A**) Sequence comparison of conserved domains. The darker blue hue indicates a higher degree of sequence conservation. (**B**) WebLogo analysis of the three conserved domains.

The conserved domains of these 99 CAD proteins are depicted in Figure 2A. To further scrutinize the structure of these domains, we employed WebLogo Version 2.8.2 for visualization (Figure 2B). The results indicated that CAD proteins universally contain three conserved domains: the NADPH-binding domain GXGGXG, Zn^1^-binding domain GHE(X)_2_G(X)_5_G(X)_2_V, and Zn^2^-binding domain GDXVGVG(X)_5_C(X)_2_C(X)_2_C(X)_7_C. Although sequence variations were observed among CAD proteins, those within the same group exhibited highly conserved sequences. Notably, despite the loss of certain sequences (Figure 2A), BolCAD7b retains two key conserved functional domains of the CAD protein family: ADH_N (NADPH-binding domain) and ADH_zinc_N (Zn^2^-binding domain). Therefore, we still considered it a member of the CAD protein family.

### 2.4. Conserved Motifs of CAD Proteins and Variations in Gene Structure in A. thaliana and the Brassica U-Triangle Species

**Figure 3:**
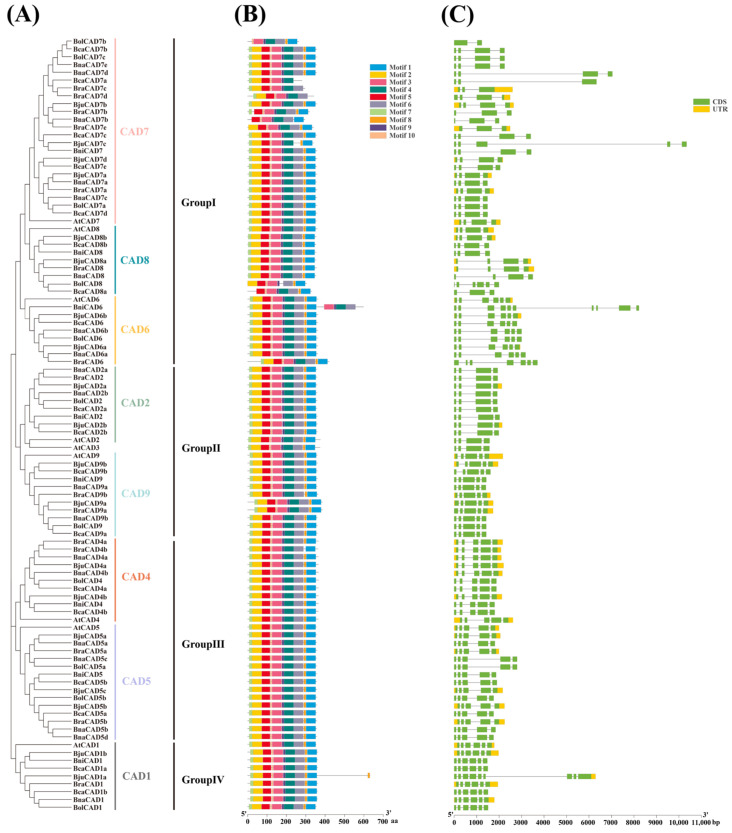
Phylogenetic tree, motif distribution, and gene structure analysis of *CADs* between *A. thaliana* and the *Brassica* U-triangle species. (**A**) Phylogenetic tree of the CAD protein family from *A. thaliana* and the *Brassica* U-triangle species. (**B**) Conserved motifs of the CAD proteins. Ten motifs are identified using the MEME program and are indicated by differently colored boxes. The black line indicates the relative length of proteins. (**C**) Gene structure of *CADs*. The green box denotes the untranslated regions (UTRs), the yellow box represents the coding sequences (CDSs), and the gray line corresponds to introns.

Based on the evolutionary relationships depicted in Figure 3A, we identified 10 conserved motifs within 99 CAD proteins using TBtools. The results revealed that 66 CAD proteins, members from subgroups CAD1, CAD2, CAD4, CAD5, CAD6, and CAD9, contained all 10 conserved motifs. In contrast, some proteins from subgroups CAD7 and CAD8 exhibited motifs loss, especially lacking Motif 1, Motif 2, Motif 7, and Motif 8 (Figure 3B). This pattern suggests that subgroups CAD7 and CAD8 have undergone greater evolutionary diversification and may exhibit more varied functions. Notably, Motif 3, Motif 9, and Motif 10 were present in all CAD proteins, indicating that these motifs constitute the core conserved structural domains of the CAD family.

We performed an exon/intron analysis of the 99 *CAD* genes. The results showed that the number of exons varied from 2 (*BolCAD7b*) to 10 (*BniCAD6*) (Figure 3C). Genes within the same subgroup generally had a similar count of exons and introns. Specifically, genes in subgroups CAD4, CAD5, and CAD9 typically contained five exons, whereas most members from subgroups CAD2, CAD7, and CAD8 had four exons, and the majority of members from subgroups CAD1 and CAD6 comprised six exons. Compared with other genes in the same subgroup, the significant deviation in exon number observed in certain genes might be attributed to gene sequence truncation, loss, duplication, or recombination events during evolutionary processes [31]. For example, *BolCAD7b* contained only two exons, fewer than the three or four exons typically found in other genes in the same subgroup. In contrast, *BjuCAD1a* had nine exons, exceeding the usual six exons observed in its subgroup counterparts. Similarly, *BniCAD6* comprised 10 exons, which was also higher than the typical number of 6 exons in other members of its subgroup.

### 2.5. Chromosomal Distribution of CAD Genes in the Brassica U-Triangle Species

**Figure 4:**
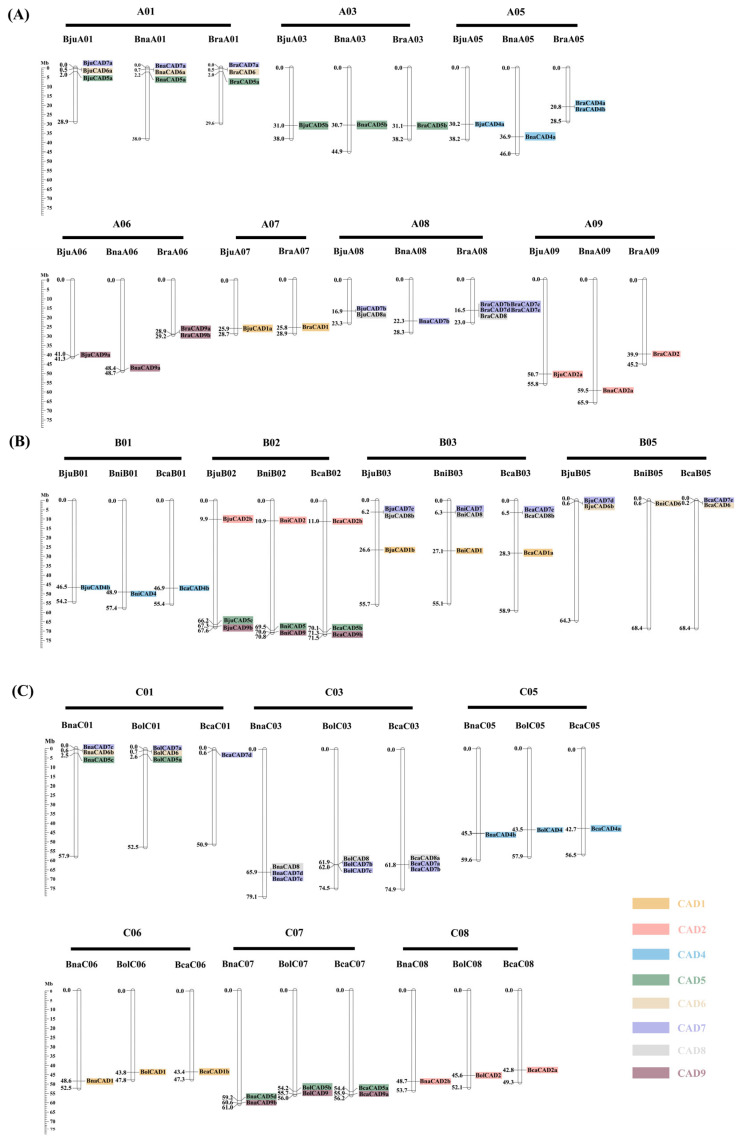
Chromosome distribution of *CAD* genes in the *Brassica* U-triangle species. (**A**) *CADs* distribution on the A subgenomes in *B. juncea*, *B. napus*, and *B. rapa*. (**B**) *CADs* distribution on the B subgenomes in *B. juncea*, *B. nigra*, and *B. carinata*. (**C**) *CADs* distribution on the C subgenomes in *B. napus*, *B. carinata*, and *B. oleracea*. The scales on the left indicate the size of the various *Brassica* chromosomes in Mb. Chromosomes within the same classification are depicted in identical colors.

There was significant diversity in the chromosomal distribution of *CAD* genes across the six species. Specifically, in *B. rapa* (AA), *CAD* genes were located on chromosomes A01, A03, A05, A06, A07, A08, and A09. In *B. nigra* (BB), *CAD* genes were found on chromosomes B01, B02, B03, and B05. In *B. oleracea* (CC), *CAD* genes were distributed across chromosomes C01, C03, C05, C06, C07, and C08. Among the allotetraploid species, *B. juncea* (AABB) contained *CAD* genes on both the A subgenome (chromosomes A01, A03, A05, A06, A07, A08, and A09) and the B subgenome (chromosomes B01, B02, B03, and B05). *B. napus* (AACC) contained *CAD* genes on the A subgenome (chromosomes A01, A03, A05, A06, A08, and A09) and the C subgenome (chromosomes C01, C03, C05, C06, C07, and C08). Finally, *B. carinata* (BBCC) had *CAD* genes located on the B subgenome (chromosomes B01, B02, B03, and B05) and the C subgenome (chromosomes C01, C03, C05, C06, C07, and C08) (Figure 4). The distribution of *CAD* genes among the subgenomes A, B, and C was relatively even, with 33, 26, and 31 *CAD* genes identified in each subgenome, respectively (Figure 4). Notably, genes occupying parallel physical positions within the same subgenome exhibited extensive collinearity across the *Brassica* U-triangle species. This observation suggests that, despite the distinct distribution patterns of *CAD* genes among different species, the chromosomal architecture of these subgenomes remains highly conserved.

### 2.6. Collinearity Analysis of CAD Genes in A. thaliana and the Brassica U-Triangle Species

**Figure 5:**
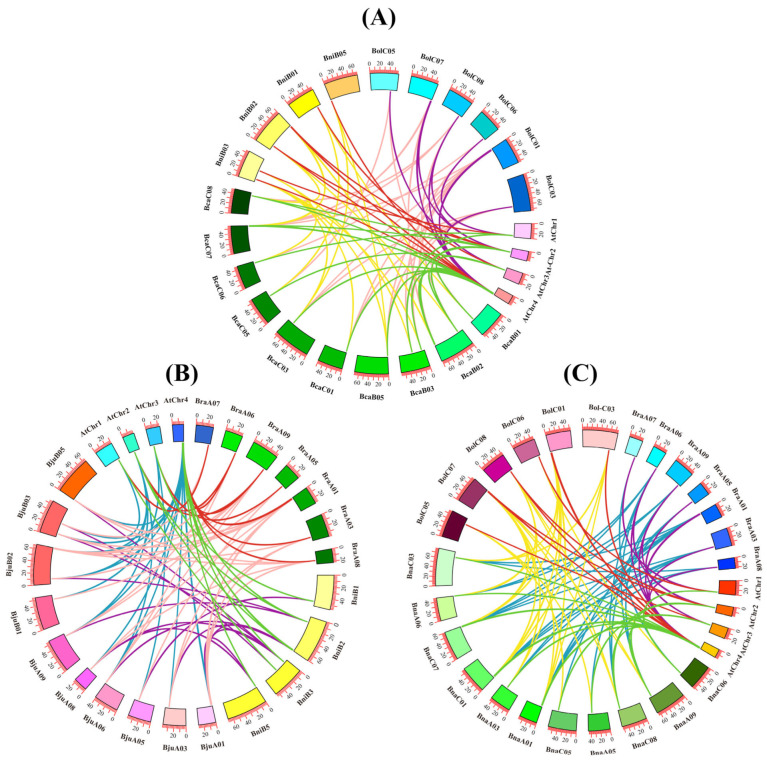
Collinearity analysis of *CAD* genes between *A. thaliana* and *Brassica* U-triangle species. (**A**) Collinearity analysis of *CAD* genes between *A. thaliana*, *B. nigra*, *B. oleracea*, and *B. carinata*. (**B**) Collinearity analysis of *CAD* genes between *A. thaliana*, *B. nigra*, *B. rapa*, and *B. juncea*. (**C**) Collinearity analysis of *CAD* genes between *A. thaliana*, *B. oleracea*, *B. rapa*, and *B. napus*. Divergent colors of connecting lines indicate distinct synteny relationships among various species.

We divided *CAD* genes into three categories based on their subgenome relationships. Each category included *A. thaliana*, an allopolyploid species, and its two diploid progenitors. Specifically, Category A comprised *A. thaliana*, *B. carinata*, *B. nigra*, and *B. oleracea*; Category B included *A. thaliana*, *B. juncea*, *B. rapa*, and *B. nigra*; Category C consisted of *A. thaliana*, *B. napus*, *B. rapa*, and *B. oleracea*. In each category, we constructed five pairwise comparisons of syntenic relationships: between *A. thaliana* and each of the two diploid progenitors, between *A. thaliana* and the allopolyploid, and between each diploid progenitor and the allopolyploid. The numbers of orthologous gene pairs varied among different species. In Category A, we identified 19 orthologous gene pairs between *A. thaliana* and *B. carinata*, 9 pairs between *A. thaliana* and *B. nigra*, 11 pairs between *A. thaliana* and *B. oleracea*, 17 pairs between *B. nigra* and *B. carinata*, and 22 pairs between *B. oleracea* and *B. carinata*. In Category B, the orthologous gene pairs included 21 pairs between *A. thaliana* and *B. juncea*, 10 pairs between *A. thaliana* and *B. rapa*, 9 pairs between *A. thaliana* and *B. nigra*, 26 pairs between *B. rapa* and *B. juncea*, and 15 pairs between *B. nigra* and *B. juncea*. In Category C, we observed 17 pairs between *A. thaliana* and *B. napus*, 10 pairs between *A. thaliana* and *B. rapa*, 11 pairs between *A. thaliana* and *B. oleracea*, 27 pairs between *B. rapa* and *B. napus*, and 29 pairs between *B. oleracea* and *B. napus* (Figure 5). Overall, we identified 86 genes with syntenic relationships among the 99 genes examined. These syntenic *CAD* gene pairs were widely distributed across the genomes of *A. thaliana* and the *Brassica* U-triangle species. Notably, the collinearity of 13 genes was found to be weak, including *BnaCAD7b*, *BolCAD6*, *BraCAD4b*, *BraCAD9b*, *BraCAD7c*, *BraCAD7d*, *BraCAD7e*, *BraCAD8*, *BjuCAD1a*, *BniCAD8*, *BcaCAD8b*, *BcaCAD6*, and *BcaCAD7a*.

**Figure 6:**
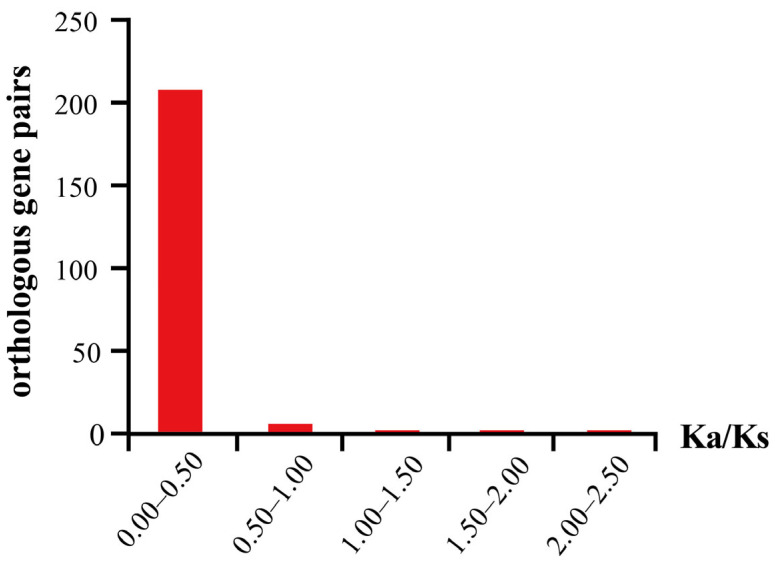
The distribution of Ka/Ks ratio between the orthologous gene pairs in *A. thaliana* and *Brassica* U-triangle species.

The Ka/Ks analysis revealed that most gene pairs were under strong purifying selection, with Ka/Ks ratios less than 1 and predominantly ranging from 0 to 0.5 (Figure 6). However, three gene pairs exhibited potential signatures of positive selection, with Ka/Ks ratios exceeding 1: *BolCAD*-*BnaCAD8* (1.14), *BraCAD6*-*BnaCAD6a* (1.58), and *BolCAD8*-*BcaCAD6a* (2.17).

### 2.7. Cis-Acting Element Analysis of BnaCAD Promoters

**Figure 7:**
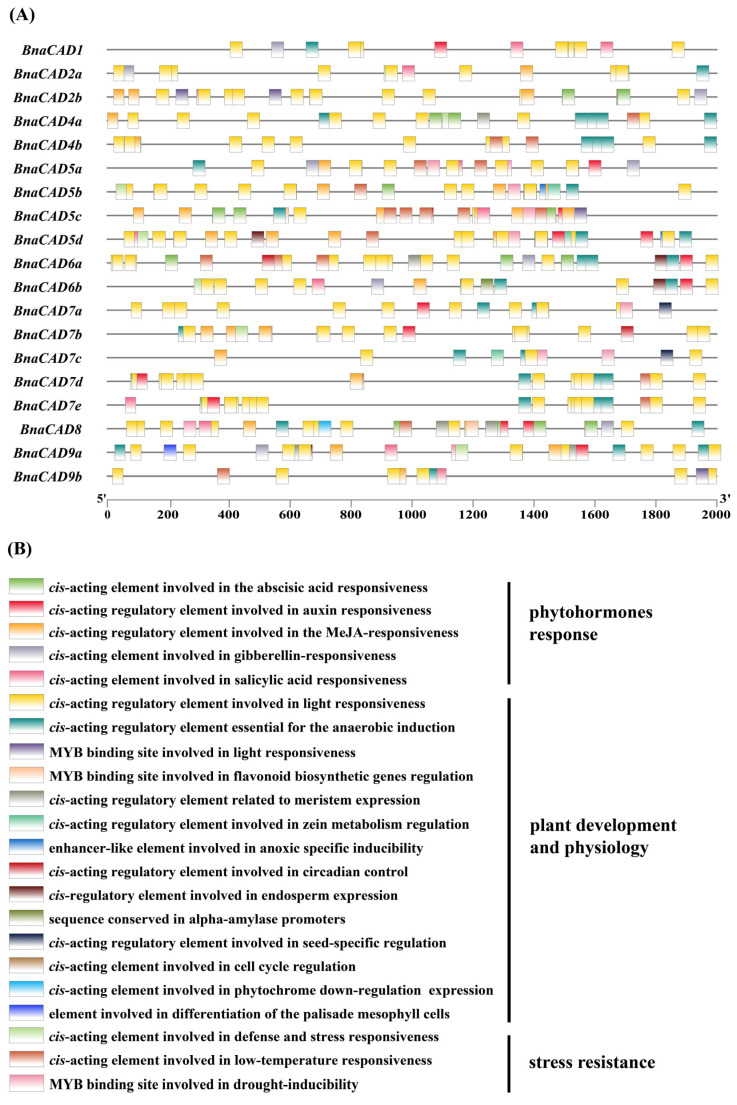
*Cis-*acting elements in the promoter regions of *BnaCADs*. (**A**) Distribution and localization of *cis*-acting elements in the promoter regions of *BnaCADs.* The scale at the bottom indicates the length of the sequence. The different *cis-*acting elements are indicated by different colors. (**B**) Functional classification of *cis*-acting elements in the promoter regions of *BnaCADs*.

Nineteen *CAD* genes have been identified in *B. napus* (Table S1), and a comprehensive analysis of *cis*-acting elements within their promoters was conducted (Figure 7A). A total of 22 distinct *cis*-acting elements were identified and categorized into three major groups based on their primary functions in transcriptional regulation. These included phytohormone-response elements (5 elements), elements associated with plant physiological and developmental processes (14 elements), and stress-resistance elements (3 elements) (Figure 7B). Furthermore, except for *BnaCAD1*, *BnaCAD6a*, *BnaCAD7a*, and *BnaCAD7e*, the promoters of the remaining 15 *BnaCAD* genes contained the MYB binding site involved in the regulation of flavonoid biosynthetic genes (Figure 7A). Flavonoids play crucial roles in pigmentation, disease resistance, and defense against biotic and abiotic stresses [32]. The presence of these regulatory elements further supports the involvement of *BnaCAD* genes in regulating seed coat color and in responding to abiotic stresses. Our study highlights the pleiotropic regulatory roles of *BnaCAD* genes in *B. napus* growth and development, stress responses, and phytohormone signaling. These findings underscore the multifaceted functions of *BnaCAD* genes in various biological processes and provide insights into their potential regulatory mechanisms.

### 2.8. Expression Profiles of BnaCAD Genes in B. napus Under Drought, Osmotic, and ABA Treatments

**Figure 8:**
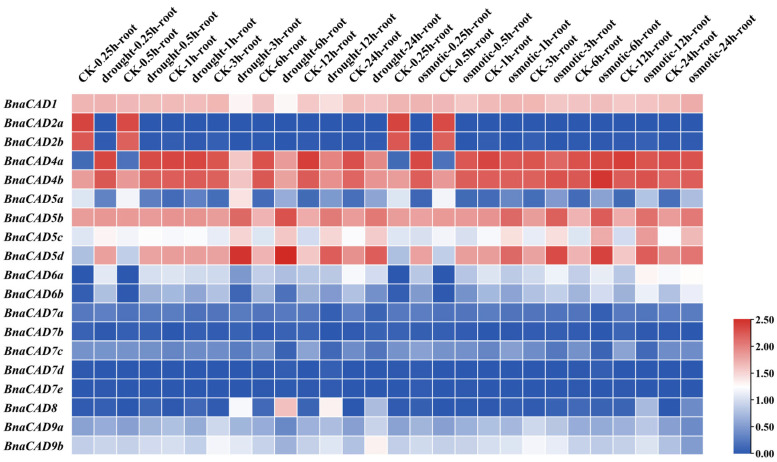
Expression profiles of *CAD* genes in *B. napus* under drought and osmotic treatments. RNA-Seq data are downloaded from BnIR database (https://yanglab.hzau.edu.cn/BnIR, accessed on 2 January 2025). The expression profiles of each *BnaCAD* were calculated as Log10 (FPKM value + 1). CK (control), no stress. The color bar on the right indicates the relative level of gene expression.

We analyzed the expression patterns of 19 *BnaCAD* genes under drought and osmotic conditions in *B. napus* cultivar ZS11 using published RNA-Seq datasets from the BnIR database (https://yanglab.hzau.edu.cn/BnIR, accessed on 2 January 2025). Specifically, during the initial phase of treatment (0.25 to 0.5 h), the expression levels of *BnaCAD2a*, *BnaCAD2b*, and *BnaCAD5a* were significantly downregulated, with FPKM (fragments per kilobase of transcript per million mapped reads) values decreasing by 10- to 300-fold. In contrast, *BnaCAD4a*, *BnaCAD4b*, *BnaCAD5d*, *BnaCAD6a*, and *BnaCAD6b* showed significant upregulation, with FPKM values increasing by 30- to 300-fold. During the subsequent period (3–24 h), *BnaCAD5a*, *BnaCAD5b*, *BnaCAD5c*, *BnaCAD5d*, and *BnaCAD8* showed noticeable upregulation (FPKM values increased by 10-fold), whereas *BnaCAD4a* and *BnaCAD4b* were downregulated (FPKM values decreased by 100-fold) (Figure 8). These results indicate that different *BnaCAD* genes exhibit distinct temporal expression patterns in response to stress. Overall, 14 of 19 *BnaCAD* genes in *B. napus* were found to be sensitive to drought and osmotic stress, while the *BnaCAD7* subgroup (*BnaCAD7a–7e*) showed no significant changes, suggesting that this subgroup may not be involved in abiotic stress responses or may function through alternative mechanisms. Among the responsive genes, the *BnaCAD2* subgroup (2a, 2b), *BnaCAD4* subgroup (4a, 4b), and *BnaCAD5* (5a, 5b, 5c, 5d) subgroup were found to play major roles. *BnaCAD2a* and *BnaCAD2b* were stably downregulated throughout the duration of stress. *BnaCAD4a* and *BnaCAD4b* exhibited a biphasic pattern, with initial upregulation followed by downregulation. Members of the *BnaCAD5* subgroup showed functional differentiation. Some members, such as *BnaCAD5b*, *BnaCAD5c*, and *BnaCAD5d*, were persistently upregulated, while *BnaCAD5a* was initially downregulated before being upregulated.

**Figure 9:**
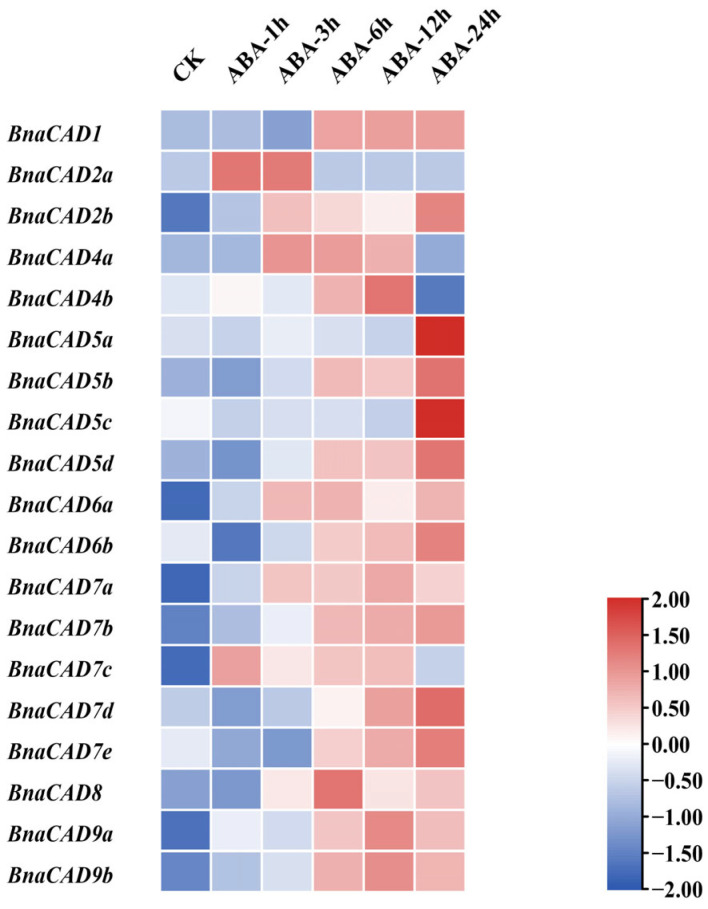
Expression profiles of *BnCAD* genes in *B. napus* under ABA treatment. RNA-Seq data are downloaded from BnaGADB v1.0 (http://www.bnagadb.cn/, accessed on 2 January 2025). The expression profile of each *BnaCAD* was calculated as Log10 (FPKM value + 1). CK (control), no stress. The color bar on the right indicates the relative level of gene expression.

Our analysis revealed that the expression levels of *BnaCAD* genes in ZS11 were significantly upregulated with increasing ABA treatment duration using published RNA-Seq data from BnaGADB v1.0 (Figure 9). However, different *BnaCAD* genes exhibited distinct response patterns. For instance, *BnaCAD2a* and *BnaCAD7c* were highly expressed during the early stages of treatment (1–3 h, 2 < FPKM < 30), whereas *BnaCAD1*, *BnaCAD2b*, *BnaCAD4a*, *BnaCAD4b*, *BnaCAD5a*, *BnaCAD5b*, *BnaCAD5c*, *BnaCAD5d*, *BnaCAD6a*, *BnaCAD6b*, *BnaCAD7a*, *BnaCAD7b*, *BnaCAD7d*, *BnaCAD7e*, *BnaCAD8*, *BnaCAD9a*, and *BnaCAD9b* were more highly expressed during the later stages of treatment (6–24 h, 2 < FPKM < 100).

### 2.9. Expression Pattern of Four BnaCAD Genes in Yellow- and Black-Seeded B. napus

**Figure 10:**
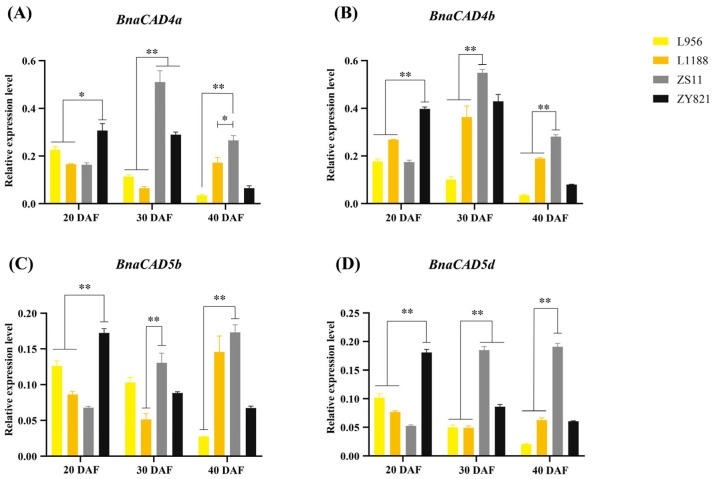
Expression pattern of four *BnaCADs* in yellow-seeded *B. napus* materials and black-seeded ones. *BnaCAD4a* (**A**), *BnaCAD4b* (**B**), *BnaCAD5b* (**C**), and *BnaCAD5d* (**D**). L956 and L1188, yellow-seeded materials; ZS11 and ZY821, black-seeded materials. DAF, days after flowering. Data of RT-qPCR were normalized to the expression level of *BnaActin7*. Values are presented as mean ± SD. Statistically significant differences were analyzed using Student’s *t*-test (*, *p* < 0.05, **, *p* < 0.01).

We confirmed that the expression levels of the four genes, including *BnaCAD4a*, *BnaCAD4b*, *BnaCAD5b*, and *BnaCAD5d*, were generally higher in black-seeded *B. napus* (ZS11 and ZY821) compared to yellow-seeded ones (L956 and L1188) at all tested developmental stages (20, 30, and 40 DAF, days after flowering). Additionally, the temporal expression patterns of these four *CAD* genes differed between the two black-seeded materials. Specifically, at 20 DAF, the expression levels of the four *CAD* genes in ZY821 were significantly higher than those in the two yellow-seeded materials (*p* < 0.05 or *p* < 0.01). At 30–40 DAF, the expression levels of these genes in ZS11 were considerably higher than those in the two yellow-seeded materials (*p* < 0.05 or *p* < 0.01, Figure 10).

## 3. Discussion

### 3.1. Expansion, Loss, and Functional Diversification of the CAD Gene Family in the Brassica U-Triangle Species

The dynamic evolution of gene families, driven by mechanisms such as whole-genome duplication (WGD) and selective gene loss, is a key strategy for plants to adapt to complex environments and shape functional diversity [33]. The evolutionary trajectory of the CAD gene family in the *Brassica* U-triangle species exemplifies this balance. Although whole-genome triplication (WGT) events theoretically result in a threefold increase in amounts of the gene members [34], our analysis revealed significant deviations. The *B. rapa* (AA), *B. nigra* (BB) and *B. oleracea* (CC) retained only 15, 8, and 11 *CAD* genes, respectively, far below theoretical amounts, namely threefold of the corresponding ones in *A. thaliana* (Table S1). This phenomenon of “post-expansion loss” aligns with subgenome dominance and selective pressure following polyploidization [3]. Notably, the number of *CAD* genes in the allotetraploid species (*B. juncea*, *B. napus*, and *B. carinata*) is not simply equal to the sum of genes in their own diploid progenitors. For instance, *B. juncea* (AABB) has 19 CAD genes, fewer than the sum of its diploid progenitors *B. rapa* (15) and *B. nigra* (8), indicating that redundant copies were pruned to optimize metabolic networks. Crucially, gene loss does not signify functional degradation. Instead, it drives innovation by retaining critical clades and promoting adaptive evolution [35].

The expansion of CAD gene families is a common feature in land plant evolution. For example, green algae possess only one single CAD homolog, whereas *A. thaliana* and *Z. mays* retain 9 and 12 members, respectively [17,26,36]. The expansion of *CAD* genes correlates with the increasing complexity of plant cell walls. They may provide plants with increased enzyme activity, which promotes lignin synthesis and cell wall reinforcement, thereby enabling plants to better adapt to environmental stress [36]. There are only two bona fide *CAD* genes in *A. thaliana* [17]. However, the number of bona fide *CAD* genes in Group III of the *Brassica* U-triangle species has increased to two to six (Table S1). This suggests a significant amplification of *CAD* genes in Group III during evolution, indicating that these genes may play a stronger role in lignification in the *Brassica* U-triangle species, which needs further validation. The Ka/Ks ratio is a crucial indicator for assessing the evolutionary forces acting on genes. A Ka/Ks ratio less than 1 indicates purifying selection (i.e., functional conservation), while a ratio greater than 1 suggests positive selection (i.e., functional innovation) [37]. In this study, we found that 90% of the *CAD* gene pairs in the U-triangle species are under purifying selection, with Ka/Ks ratios less than 1 and predominantly within the 0–0.5 range. This finding indicates that their core functions, such as lignin synthesis, are highly conserved during evolution. However, three gene pairs (*BolCAD8-BcaCAD6a*, *BraCAD6-BnaCAD6a*, and *BolCAD-BnaCAD8*) exhibited significant signals of positive selection, with Ka/Ks ratios greater than 1.14 and even up to 2.17, implying that they have undergone adaptive evolution in specific habitats (Figure 6). This selective pressure may drive functional innovation, such as some copies shifting toward stress response while others maintain the conserved function of lignin synthesis (Figure 8). These results demonstrate that gene loss and expansion are not opposing processes but rather jointly shape functional diversity through pruning redundancy and strengthening core functions.

### 3.2. Transcriptional Regulation of BnaCAD Genes in Stress Responses and Seed Coat Color Formation

The *cis*-acting elements in promoters are fundamental transcriptional regulatory units that play crucial roles in regulating gene expression associated with numerous biological processes and stress responses [38]. For instance, ABA-responsive elements (ABREs) activate downstream stress-responsive genes via the SnRK2-AREB/ABF signaling cascade [39]. In this study, we identified four phytohormone-responsive *cis*-acting elements (ABA, auxin, MeJA, and gibberellin) and stress-resistance *cis*-acting elements in the promoters of *BnaCAD* genes (Figure 7). It suggests that the expression of *BnaCAD* genes may be regulated by multiple signaling pathways involved in both hormone responses and stress adaptation. It is worth noting that ABREs were widely distributed across 13 *BnaCAD* genes. During the late phases of drought and osmotic stress, the expression levels of *BnaCAD5b*, *BnaCAD5c*, *BnaCAD5d*, and *BnaCAD8* were significantly upregulated (Figure 8), consistent with their differential expression patterns under ABA treatment (Figure 9). This co-regulation suggests that these genes may enhance stress tolerance through ABA-mediated pathways, potentially via SnRK2-AREB/ABF signaling cascades that activate downstream stress-responsive targets, which needs further validation. In contrast, *BnaCAD4a* and *BnaCAD4b* exhibited a distinct expression trend under drought and osmotic stress, with initial upregulation followed by downregulation (Figure 8). Such divergence implies that they may regulate stress response through other pathways independent of ABA-mediated pathways. These findings highlight the functional diversification of *BnaCAD* genes, where specific members adopt distinct transcriptional strategies to optimize adaptive responses under fluctuating environmental conditions. Flavonoids, as secondary metabolites, play crucial roles in plant pigmentation and resistance against biotic and abiotic stresses [32]. For instance, they are involved in the color formation of yellow seed coats in *B. napus* [40]. Previous studies have reported a strong negative correlation between lignin content and seed coat color, which may be attributed to the fact that the lignin and flavonoid biosynthesis pathways share the same substrate [41,42,43]. In the *Brassicaceae* family, less lignin deposition is closely associated with lighter seed coat pigmentation [11], with *B. napus* serving as a prime exemplar where lignin has been shown to play a crucial role in the formation of yellow-seeded phenotypes [12]. In this study, some MYB binding sites known to be associated with flavonoid biosynthesis were identified in the promoters of most *BnaCAD* genes (Figure 7), suggesting that *BnaCAD* genes may play a potential role in the flavonoid pathway. Meanwhile, the expression of four bona fide *BnaCAD* genes (*BnaCAD4a*, *BnaCAD4b*, *BnaCAD5b,* and *BnaCAD5d*) was significantly lower in yellow-seeded materials compared to black-seeded ones. This suggests that they may influence seed coat color through the regulation of lignin synthesis (Figure 10). Therefore, future research should explore the interaction between lignin and flavonoid metabolism to comprehensively unravel the molecular mechanisms underlying seed coat color formation influenced by *BnaCAD* genes.

It has been widely reported that some transcription factors modulate the expression of genes in the phenylpropanoid pathway in response to light signals [44,45]. For example, the MYB75/PAP1 transcription factor, which responds to blue and red light, is involved in anthocyanin biosynthesis in *A. thaliana* [46]. Similarly, the blue-light signal can influence the biosynthesis of lignin by transcription factor in the stone cells of pear fruits [47]. In this study, it was found that the promoter regions of all *BnaCAD* genes contain light-responsive *cis*-acting elements. It suggests that light may influence the biosynthesis of lignin or flavonoids by regulating the expression of these genes, thereby modulating the color of the seed coat (Figure 7). Future research could analyze the dynamic changes of lignin and flavonoid metabolites in yellow- and black-seeded materials under different light conditions to decipher the mechanism by which light signals allocate metabolic fluxes.

Although this study has elucidated the evolutionary patterns and functional diversification of the CAD gene family, direct functional validation remains a limitation. Future research will address this gap by confirming the functions of these genes through CRISPR/Cas9 gene-editing technology or other techniques and will further investigate their regulatory networks in seed coat pigmentation and abiotic stress responses in *B. napus*.

## 4. Materials and Methods

### 4.1. Plant Materials and Growth Conditions

The *B. napus* materials used in this study comprised two black-seeded cultivars, Zhongshuang11 (ZS11) and Zhongyou 821 (ZY821), and two yellow-seeded accessions, L956 and L1188. These materials were cultivated at the experimental base at Southwest University in Chongqing, China. Five healthy and robust plants were randomly selected from each line for experimentation. The flowering time was marked using colored wool threads for collecting the seeds at various developmental stages. Immediately upon collection, the seeds were immersed in liquid nitrogen and stored at −80 °C for subsequent analyses.

### 4.2. Identification and Annotation of CAD Family Genes

The genome information of five U-triangle species (*B. rapa* cv. Chiifu V3.5, *B. nigra* cv. NI100 v2, *B. oleracea* cv. JZS v2, *B. juncea* cv. tum V2.0, *B. napus* cv. ZS11 HZAU V1.0) was obtained from the *Brassica* Database (BRAD, http://brassicadb.cn, accessed on 8 October 2024) and the *B. napus* multi-omics information resource database (BnIR, https://yanglab.hzau.edu.cn/BnIR, accessed on 8 October 2024) [48]. The genome data of *B. carinata* was retrieved from the NCBI Blastp program (https://blast.ncbi.nlm.nih.gov/Blast.cgi?PROGRAM=blastp&PAGE_TYPE=BlastSearch&LINK_LOC=blasthome, accessed on 8 October 2024) [49]. The amino acid sequences of nine CAD proteins from *A. thaliana* (AT1G72680, AT2G21730, AT2G21890, AT3G19450, AT4G34230, AT4G37970, AT4G37980, AT4G37990, and AT4G39330) were retrieved from the TAIR database (https://www.arabidopsis.org/, accessed on 8 October 2024) and used as queries. TBtools-BLAST (v2.154) [50] and NCBI Blastp program (https://blast.ncbi.nlm.nih.gov/Blast.cgi?PROGRAM=blastp&PAGE_TYPE=BlastSearch&LINK_LOC=blasthome, accessed on 11 October 2024) were employed to identify the CAD family proteins with the default parameters. The Conserved Domain Database (CDD) [51] from the National Center for Biotechnology Information (NCBI, https://www.ncbi.nlm.nih.gov/Structure/cdd, accessed on 11 October 2024) was used to examine conserved domains of CAD proteins. Additionally, TBtools-BatchSMART was used for visualizing domains. Tbtools-Protein Parameter Calc was used to predict the length (number of amino acid residues), molecular weight (MW, in kDa), isoelectric point (pI), and stability index of each CAD protein. The subcellular locations of proteins were predicted using the Plant Cell-PLoc 2.0-mPLoc (http://www.csbio.sjtu.edu.cn/bioinf/plant-multi/, accessed on 18 October 2024) [52].

### 4.3. Comparative Sequence Alignment and Evolutionary Divergence Assessment of CAD Family Genes

To visualize the conserved domains within CAD protein sequences, Jalview 2 was used for multiple sequence alignment (http://www.jalview.org/, accessed on 27 October 2024) [53]. Subsequently, sequence logos for the three conservative regions were generated using WebLogo Version 2.8.2 (http://weblogo.berkeley.edu/logo.cgi, accessed on 27 October 2024) [54].

The evolutionary relationship and divergence among proteins were inferred through phylogenetic analysis. The phylogenetic tree was constructed using MEGA 11 (Tokyo Metropolitan University, Tokyo, Japan) with the following parameters: MUSCLE alignment, Jones–Taylor–Thornton (JTT) model, Bootstrap method with 1000 replicates, and partial deletion threshold at 80% [55]. The tree was then refined and visualized using Evolview (https://www.evolgenius.info/evolview/#/, accessed on 26 October 2024) [56].

### 4.4. Functional Domain Conservation and Genomic Architecture Profiling in CAD Family Genes

The Multiple Expectation Maximization for Motif Elucidation (MEME) program (version 5.5.0) was employed to identify conserved motifs present within CAD proteins [57]. The MEME Suite is accessible online at https://meme-suite.org/meme/doc/meme.html (accessed on 29 October 2024). Subsequently, the identified motif structures, gene structures, and evolutionary relationships were visualized using the TBtools software [49], available at https://github.com/CJ-Chen/TBtools (accessed on 29 October 2024).

### 4.5. Chromosomal Distribution and Colinearity Analysis of CAD Genes

The General Feature Format (GFF) genome files of the U-triangle species were obtained from the *Brassica napus* multi-omics information resource database (BnIR) (available at https://yanglab.hzau.edu.cn/BnIR, accessed on 2 November 2024) [48]. The chromosomal location information of the *CAD* family genes was extracted from these files. Subsequently, MapChart V2.32 was employed to map the *CAD* family genes onto their corresponding chromosomes and to visualize their distribution [58]. To identify gene duplication patterns and perform collinearity analysis, TBtools-One Step MCScanX and TBtools-Amazing Super Circos were utilized. Additionally, the nonsynonymous substitution rate (Ka) and synonymous replacement rate (Ks) were calculated, and the selective pressures on gene evolution were assessed using the Ka/Ks ratio [37].

### 4.6. Cis-Element Analysis of BnaCAD Promoters

To elucidate the *cis*-acting elements of the *BnaCAD* genes, we employed the TBtools-Gtf/GFF3 Sequences Extract tool to retrieve 2000 bp upstream sequences of these genes. Subsequently, these sequences were submitted to the Plant CARE database (http://bioinformatics.psb.ugent.be/webtools/plantcare/html/; accessed on 2 January 2025) for the prediction of putative *cis*-acting elements [59]. The identified elements were then visualized using the Tbtools-Simple BioSequence Viewer.

### 4.7. Expression Profile Analysis of BnaCAD Genes

To elucidate the roles of *CAD* genes in response to abiotic stress and hormone treatment in *B. napus*, we analyzed public RNA-Seq datasets. Specifically, we obtained RNA-Seq data for drought and osmotic stress treatments from the *Brassica napus* Integrated Resource (BnIR) database (https://yanglab.hzau.edu.cn/BnIR, accessed on 2 January 2025) [48]. Additionally, we retrieved the RNA-Seq data for ABA treatment of ZS11 from BnaGADB v1.0 (http://www.bnagadb.cn/, accessed on 2 January 2025). The relative expression levels of the genes were normalized using the Log10 (FPKM value + 1) method, and heatmaps were generated with TBtools to visualize the expression patterns of *BnaCAD* genes [60].

Among the six bona fide *CAD* genes identified in *B. napus* (Table S1), four highly expressed *CAD* genes, including *BnaCAD4a*, *BnaCAD4b*, *BnaCAD5a*, and *BnaCAD5b*, were selected for further investigation into their roles in regulating seed coat color. These selections were based on published RNA-Seq data from the BnIR database. We used RT-qPCR to analyze the expression profiles of these genes in seeds of two yellow-seeded materials (L956, L1188) and two black-seeded materials (ZS11, ZY821) at 20, 30, and 40 DAF (days after flowering). Total RNA was extracted from the seeds at different developmental stages using an EZ-10 DNAaway RNA Mini-Prep Kit (Sangon, Shanghai, China) according to the manufacturer’s instructions. The extracted RNA was reverse-transcribed into complementary DNA (cDNA) using ExonScript RT Mix (with dsDNase) kit (Baoguang, Chongqing, China). Subsequently, quantitative real-time PCR (qPCR) was performed in triplicate on a Bio-Rad CFX96 Real-Time System (Bio-Rad Laboratories, Hercules, CA, USA) using SYBR Premix Ex Taq II (Takara, Dalian, China). The relative expression levels were calculated using the 2^−∆∆CT^ method [61], with *BnaActin7* as the internal standard. The primers used in this study are listed in Table S2.

## 5. Conclusions

This study provides a comprehensive characterization of the CAD gene family in the *Brassica* U-triangle species, identifying 90 *CAD* genes and elucidating their roles in lignin biosynthesis, abiotic stress adaptation, and seed coat pigmentation. The findings offer valuable insights for breeding *B. napus* cultivars with enhanced oil quality and stress resilience, potentially improving the productivity under environmental constraints. Future work will focus on validating the functions of key *CAD* genes using CRISPR/Cas9 and exploring the interplay between lignin and flavonoid metabolism to further optimize seed traits and stress responses.

## Data Availability

All additional datasets supporting the findings of this study are included within the article and Supplementary Materials.

## Supplementary Materials

The following supporting information can be downloaded at: https://www.mdpi.com/article/10.3390/plants14081184/s1, Table S1: Characteristics of CADs from the *Brassica* U-triangle species.; Table S2: Primers used for RT-qPCR analysis of four *BnaCAD* genes.
